# Psychosocial impacts of the COVID-19 pandemic among settled women: A longitudinal study

**DOI:** 10.1590/1518-8345.6123.3831

**Published:** 2023-03-06

**Authors:** Jaqueline Lemos de Oliveira, Janaina Cristina Pasquini de Almeida, Antonio Jose Correa de Pauli, Mara Regina Moitinho, Regina Célia Fiorati, Jacqueline de Souza

**Affiliations:** 1 Universidade de São Paulo, Escola de Enfermagem de Ribeirão Preto, PAHO/WHO Collaborating Centre for Nursing Research Development, Ribeirão Preto, SP, Brazil.; 2 Scholarship holder at the Coordenação de Aperfeiçoamento de Pessoal de Nível Superior (CAPES), Brazil.; 3 Centro Nacional de Pesquisa em Energia e Materiais, Laboratório Nacional de Biorrenováveis, Campinas, SP, Brazil.

**Keywords:** COVID-19, Women, Mental Health, Social Vulnerability, Rural Population, Longitudinal Studies, COVID-19, Mulheres, Saúde Mental, Vulnerabilidade Social, População Rural, Estudos Longitudinais, COVID-19, Mujeres, Salud Mental, Vulnerabilidad Social, Población Rural, Estudios Longitudinales

## Abstract

**Objective:**

to analyze the psychosocial impacts of the COVID-19 pandemic among Brazilian women from rural settlements.

**Method:**

this is a quantitative and longitudinal study conducted with 13 settled women. The data were collected between January 2020 and September 2021 using questionnaires on the perception of the social environment (quality of life, social support, self-efficacy), common mental disorder symptoms and sociodemographic aspects. The data were analyzed using descriptive statistics, cluster analysis and variance analysis.

**Results:**

intersecting vulnerability conditions were identified that possibly intensified the challenges arising from the pandemic. The Quality of Life physical domain fluctuated differently and inversely according to the mental disorder symptoms. As for the psychological domain, at the end of the segment, an increase over time was identified in the entire sample, as the women’s perception was better than before the pandemic.

**Conclusion:**

worsening of the participants’ physical health deserves to be highlighted and, probably, it can be related to the difficulty accessing health services in this period as well as to the fear of contamination. Despite this, the participants were emotionally resilient throughout the period, including signs of improvement in terms of psychological aspects, suggesting a possible effect of the community organization of the settlement.

## Resumo

**Objetivo:**

analisar os impactos psicossociais da pandemia de COVID-19 entre mulheres brasileiras de assentamentos rurais.

**Método:**

trata-se de um estudo quantitativo longitudinal com 13 mulheres assentadas. Os dados foram coletados entre janeiro de 2020 e setembro de 2021 utilizando questionários sobre a percepção do ambiente social (qualidade de vida, apoio social, autoeficácia), sintomas de transtorno mental comum e aspectos sociodemográficos. Os dados foram analisados por meio de estatística descritiva, análise de agrupamento e de variância.

**Resultados:**

foram identificadas condições de vulnerabilidade interseccionadas que, possivelmente, exacerbaram os desafios decorrentes da pandemia. O domínio físico da qualidade de vida oscilou diferentemente e inversamente de acordo com os sintomas de transtorno mental. Quanto ao domínio psicológico, no final do segmento, identificou-se em toda a amostra um incremento ao longo do tempo, pois a percepção das mulheres estava melhor do que antes da pandemia.

**Conclusão:**

a piora na saúde física das participantes merece destaque e, provavelmente, pode estar relacionada à dificuldade de acesso aos serviços de saúde neste período bem como ao medo da contaminação. Apesar disso, as participantes apresentaram-se emocionalmente resilientes em todo o período, inclusive, com sinais de melhora em relação aos aspectos psicológicos, sugerindo um possível efeito da organização comunitária do assentamento.

## Resumen

**Objetivo:**

analizar los impactos psicosociales de la pandemia de COVID-19 en mujeres brasileñas de asentamientos rurales.

**Método:**

se trata de un estudio longitudinal cuantitativo con 13 mujeres asentadas. Los datos se recolectaron entre enero de 2020 y septiembre de 2021 mediante cuestionarios sobre percepción del entorno social (calidad de vida, apoyo social, autoeficacia), síntomas de trastorno mental común y aspectos sociodemográficos. Los datos se analizaron mediante estadística descriptiva, análisis de conglomerados y análisis de varianza.

**Resultados:**

se identificaron condiciones de vulnerabilidad entrelazadas que posiblemente exacerbaron los desafíos impuestos por la pandemia. El dominio físico de la calidad de vida fluctuó de manera diferente e inversa de acuerdo a los síntomas del trastorno mental. En cuanto al dominio psicológico, al final del segmento, se identificó un aumento en el tiempo en toda la muestra, dado que la percepción de las mujeres era mejor que antes de la pandemia.

**Conclusión:**

hay que destacar el empeoramiento de la salud física de las participantes que, probablemente, puede estar relacionado con la dificultad para acceder a los servicios de salud durante ese período y el miedo al contagio. A pesar de eso, las participantes fueron resilientes emocionalmente durante todo el período e, inclusive, tuvieron signos de mejoría en los aspectos psicológicos, lo que sugiere un posible efecto de la organización comunitaria del asentamiento.

**Highlights**

(1) Women in rural settlements are permeated by intersecting vulnerabilities.

(2) Worsening in the quality of physical health was one of the main impacts of the pandemic.

(3) Settled women proved to be psychologically resilient during this period.

(4) There was an inverse relationship between psychological symptoms and quality of the community environment.

## Introduction

Prioritization of people in situations of social vulnerability is a recommendation in force in national and international health agendas and takes on even greater importance in the current COVID-19 pandemic context, both because they present a greater risk of illness and death and because of the social and health repercussions inherent to this process^1^ ^-^ ^8^.

Women are also an important group to be considered in terms of public policies and of programs in the health and social protection areas, in view of the inequality and gender violence that mark society in most countries of the world and their repercussions in terms of health and quality of life^9^.

Recent studies, which approach gender inequality and poverty as overlapping aspects, have considered the specificities of different subgroups of women, namely: pregnant women; lactating women; women who have been victims of violence and those with any clinical condition or morbidity^1^ ^,^ ^3^ ^,^ ^10^ ^-^ ^12^.

Among these subgroups, women who live in rural settlements deserve to be highlighted given the precarious infrastructure that generally characterizes these places, as well as the difficult access to essential resources such as health, education, basic sanitation and transportation^13^ ^-^ ^15^. In addition to that, rural work, intensely and socially discredited and typical of this manual labor population, is added to the exercise of other roles culturally attributed to women, culminating in overload and intensifying the risks of worse living and health conditions in this group^13^ ^-^ ^15^.

Studies carried out in settlements from Kenya and India during the pandemic period reported an increase in the burden of poverty, discrimination, violence and depressive and anxiety-related symptoms in this population, in addition to the excessive work of women, which also includes non-paid care activities^13^ ^,^ ^16^. In Brazil, a study found that settled women experience more distress in relation to issues such as precarious housing, itinerancy, social isolation and silencing^14^.

Recent studies on the repercussions of the pandemic on settled women have mainly emphasized the socioeconomic consequences and symptoms of mental disorders as outcome variables^13^ ^-^ ^16^. Therefore, there is a need to expand the body of knowledge about the impacts of the pandemic, also considering more subjective, complex and relational aspects of the mental health of such women. Thus, the objective of the current study was to analyze the psychosocial impacts of the COVID-19 pandemic among Brazilian women from rural settlements.

## Method

### Study design, locus and period

This is a quantitative and longitudinal study developed based on the Strengthening the Reporting of Observational Studies in Epidemiology (STROBE) initiative checklist^17^. It was carried out in a settlement from the municipality of Ribeirão Preto, inland São Paulo, from January 2020 to September 2021.

In general, rural settlements in Brazil result from the tension between the Landless Workers Movement (*Movimento de Trabalhadores Sem Terra*, MST) and the federal agrarian reform agency (*Instituto Nacional de Colonização e Reforma Agrária*) with a view to the occupation of unproductive land by rural workers, considering the fundamental right of access to land^18^. The subsistence culture, based on the agroforestry system and on preservation of the environment, is a basic premise of this movement and is added to the possibility of direct commercialization of products to the population, without intermediation of large traders, in opposition to the current logic of obtaining exaggerated profits^18^. Considering that the agrarian reform is not a priority agenda for most federal administrations, the issue of social (health, education, transportation, housing) and productive (technical assistance, machinery, capital) infrastructure is quite precarious in most Brazilian rural settlements^19^, in addition to being a characteristic of the settlement under study. With approximately 470 families settled in 1,541 hectares of land, the referred place generally comprises families with low socioeconomic conditions and who live in wooden houses without access to basic sanitation (piped water, sewage system), some covered with tarpaulins and distant from most of the basic resources that are concentrated in the urban area of the city.

### Population and sample

The study population consisted of settled women, over 18 years of age and users registered at the settlement’s health unit. The estimated population under responsibility of this unit is 3,744 inhabitants, of which approximately 1,910 are women, with 1,046 over 18 years of age. 

The recruitment process was carried out through posters and pamphlets posted in the waiting room and offices, as well as via a direct approach carried out in home visits or in the waiting room of the aforementioned unit. The face-to-face approach was performed with 106 women, of which eight were excluded due to the clinical condition that precluded their participation and 45 refused the invitation, citing lack of time, lack of interest in the topic and concern about missing the appointment.

**Figure 1:**
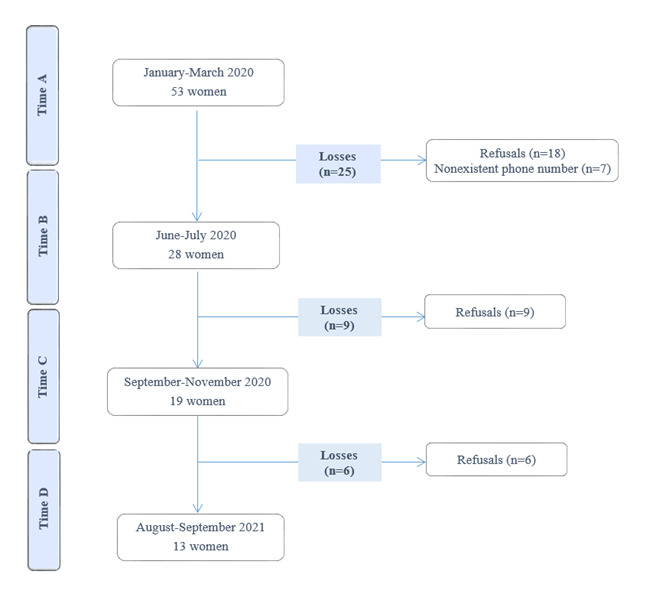
Flowchart of the number of participants (n=13). Ribeirão Preto, SP, Brazil, 2020-2021

Thus, considering the women who accepted the invitation to participate, the convenience sample had 53 women in the first follow-up phase, as shown in Figure 1. In the second, 28 women; in the third, 19 and in the fourth and last phase, 13, constituting the final sample of the current research. 

### Data collection 

In the first place, it should be noted that in Brazil, specifically in the state of São Paulo, during the pandemic period, the government decreed a quarantine with the specification of services that would or would not be allowed to work, as well as definition of criteria for classifying phases according to the number of people infected by COVID-19, hospitalizations and deaths, namely: phase 1 - red (high alert); phase 2 - yellow (control); phase 3 - yellow (flexibilization); phase 4 - green (partial opening) and phase 5 - blue (controlled normality)^20^. 

**Figure 2:**
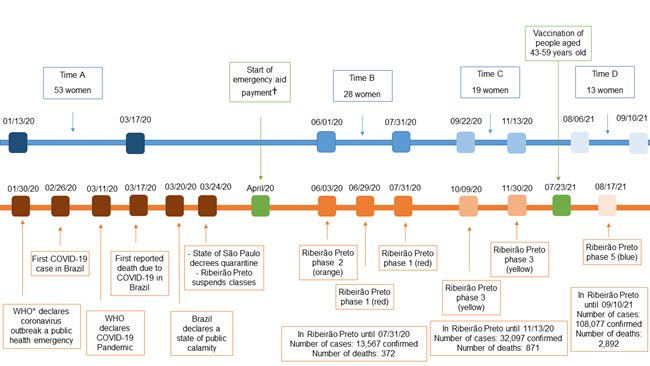
Follow-up time line with quantification of the sample and pandemic phases (n=13). Ribeirão Preto, SP, Brazil, 2020-2021

^*^WHO = World Health Organization; ^†^Emergency aid = 18 installments in Reais (R$), from R$ 300.00 to R$ 600.00 (in April/2020 equivalent to 63.34 US$ and 126.68 US$, respectively), paid to the families in socioeconomic vulnerability conditions

In view of the above, as shown in Figure 2, the data collection follow-up took place in four stages: the first, between January 13^th^ and March 17^th^, 2020 (pre-pandemic); the second, between June 1^st^ and July 31^st^, 2020 (red phase); the third, between September 22^nd^ and November 13^th^, 2020 (yellow phase) and the fourth, between August 6^th^ and September 10^th^, 2021 (blue phase).

The first phase was carried out in person at the health unit of the settlement. The others were conducted through telephone calls due to the restrictions in force during the period. Data collection was performed by a nurse with graduate level, who received training in application of the instruments listed in this research. 

A questionnaire to screen the socioeconomic profile and the following instruments were used: Quality of Life Assessment abbreviated version (WHOQOL-bref); Sarason Social Support Questionnaire; Self-Report Questionnaire - SRQ-20 and the Perceived General Self-Efficacy Scale (PGSES).

The abbreviated version of the Quality of Life Assessment instrument (WHOQOL-bref) consists of 26 questions, of which two are overall Quality of Life (QoL) questions and the others represent each of the 24 facets divided into four domains: 1-physical; 2-psychological; 3-social relationships and 4-environment. The questions are evaluated using a Likert-type scale, in which each question is scored from 1 to 5. The instrument has three questions with inverted scores and the final value results in a scale from 4 to 20, which can be transformed into a scale from 0 to 100 through the *syntax* developed by the authors^21^. The higher the score, the better the QoL perception^21^. The validated Portuguese version met the criteria for internal consistency (Cronbach’s alpha from 0.6921 to 0.9054 in the domains), in addition to those for discriminant, concurrent, content and test-retest reliability (Cronbach’s alpha from 0.69 to 0.81 in the domains)^21^. 

The Sarason Social Support Questionnaire (SSQ) consists of 27 questions, which mention the different situations in which social support can be important for an individual. The respondents must list up to nine people per question and rate their satisfaction level with the support in their respective situations. The mean of the scores through the questions measures the size of the SSQ-N network and the level of satisfaction with the support - SSQ-S^22^. In Brazil, the results for validation indicated favorable test-retest reliability and a high internal consistency level, similarly and coherently with the data provided in the scale elaboration study^22^. In the current study, the short version of the instrument, SSQ-6, was used, consisting of six items, which can be considered a practical alternative to the full version, including items related to acceptance, affection and affirmation of personal value^22^.

The Self-Report Questionnaire - SRQ-20 was developed by the World Health Organization to assess Common Mental Disorders (CMD) in developing countries^23^. The original version has 24 items, the first 20 of which are evaluators for non-psychotic disorders and four items for psychotic disorders. In the Brazilian version, as its adaptation was carried out in a Primary Health Care context, only the first 20 items are used^23^. The questionnaire has 20 “yes” or “no” questions about emotional and physical symptoms associated with psychiatric conditions. Suspected cases of mental disorder are those in which there are more than eight positive answers^23^. Sensitivity was 83% and specificity, 80%.

The Perceived General Self-Efficacy Scale (PGSES) assesses the general sense of self-efficacy perceived by the individual, aiming to predict coping with daily difficulties, as well as adaptation after experiencing stressful life events^24^. It is a Likert-type scale with ten items on a scale from 1 to 4. The total score is calculated by adding up all items, varying from 10 to 40, with higher scores indicating greater self-efficacy^24^. The scale was validated in Brazil in a study carried out with 283 individuals with a mean age of 22 years old (Cronbach’s alpha: 0.81)^24^.

### Data analysis

The data were analyzed using descriptive statistics to show the socioeconomic profile of the population. To identify the factors associated with the manifestation of symptoms of mental disorders, exploratory multivariate cluster analysis by the hierarchical method and principal components was used.

The cluster analysis grouped the sample units into groups (Group 1 and Group 2), so that there could be homogeneity within each group and heterogeneity between them. The group structure contained in the data was seen in a graph called dendrogram, prepared with the similarity matrix between the samples. The similarity matrix was constructed using the Euclidean distance and the connection of groups was performed using Ward’s method. Based on this analysis, the existence of two groups was verified, observed for the four times under study (A, B, C and D).

Subsequently, in order to determine possible characteristics that could identify similar and dissimilar behaviors in the variables adopted, a principal components multivariate analysis was chosen. For selection of the variables that would remain in the analysis, the non-collinearity criterion was used, considering for the interpretation those that presented correlation coefficients with absolute values above 0.60. The eigenvectors (principal components) were constructed based on the covariance matrix eigenvalues^25^.

To interpret the meaning of each principal component, the sign and the relative dimension of the coefficients of the linear functions were considered as an indication of the weight to be attributed to each variable. After standardizing the data (null mean and unit variance), the first two principal components (PC1 and PC2), whose eigenvalues were above 1 according to the criterion established in a previous study^26^, were considered in this study.

In order to corroborate the influence of time on the behavior of the group identified in the multivariate analysis performed previously, Analysis of variance (ANOVA) with repeated measures was undertaken. For ANOVA with repeated measures, two factors were considered: the group factor (Group 1 and Group 2) and the time factor (A, B, C and D). All the analyses were processed in the R software^27^.

Simultaneously with the statistical analyses, the basic assumptions of ANOVA, normality of errors and homogeneity of variances were tested for all variables. After performing the Shapiro-Wilk test, the data that did not present normality were transformed and, after such transformation, all data showed normality (p>0.05). p>0.05 was obtained in the Levene test to verify homogeneity of variances and, subsequently, Tukey’s test was performed to compare the means.

### Ethical aspects

The project was submitted to the Research Ethics Committee of the Ribeirão Preto Nursing School at the University of São Paulo and approved under Opinion No. 210 of September 26^th^, 2019. Development of the research followed the guidelines and regulatory standards for research involving human beings, as established by Resolution No. 466/2012 of the National Health Council.

## Results

### Characteristics of the participants 

Regarding the participants’ sociodemographic profile, it is noted that most of them were at least 40 years old (median of 49, minimum of 19 and maximum of 59), self-declared as belonging to the black or mixed races, not living in a stable union and earning monthly family incomes of up to two minimum wages (Table 1). The median number of children was 2 (minimum of 0 and maximum of 4). Two women had no diagnosed chronic diseases. In the period analyzed, four of them tested positive for COVID-19 and three did not receive emergency aid (Table 1).

**Table 1:** Distribution of the participants according to the sociodemographic characteristics (n=13). Ribeirão Preto, SP, Brazil, 2020-2021

Sociodemographic characteristics	n(%)
*Age*	
18-30 years old	04(30.8)
31-50 years old	03(23.1)
51+ years	06 (46.2)
*Skin color*	
White or Yellow	04(30.8)
Black or Brown	09(69.2)
*Religion*	
Catholic or other	03(23.1)
Protestant or Evangelical	10(76.9)
*Stable union*	
Yes	06(46.2)
No	07(53.8)
*Children*	
No children	02(15.4)
One or two	06(46.1)
Three or more	05(38.5)
*Schooling*	
Elementary School	05(38.5)
High School	07(53.8)
Higher Education	01(7.7)
*Monthly family income*	
Less than 1 MW*	05(38.5)
From 1 to 2 MWs*	06(46.2)
From 2 to 5 MWs*	02(15.4)
*Paid work*	
Yes	04(30.8)
No	09(69.2)
*Chronic diseases*	
Mental disorder	04(30.8)
Others	07(53.8)
No diagnosis	02(15.4)
*Positive COVID-19 test*	
Yes	04(30.8)
No	09(69.2)
*Received emergency aid*	
Yes	10(76.9)
No	03(23.1)

*MW = Minimum wage in force in 2020 (R$ 1,045.00, equivalent to US$ 220.64)

### CMD symptoms and perception of the social environment over the period under study

**Figure 3:**
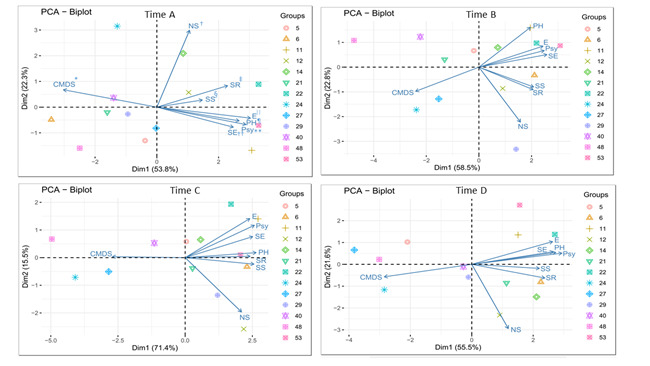
Distribution of the participants according to the psychosocial indicators analyzed (n=13). Ribeirão Preto, SP, Brazil, 2020-2021

^*^CMDS = Common Mental Disorder Symptoms; ^†^NS = Number of Supporters; ^‡^SR = Social Relationships domain; ^§^SS = Satisfaction with the Support; ^||^E = Environment domain; ^¶^PH = Physical domain; ^**^Psy = Psychological domain; ^††^SE = Self-Efficacy

Considering the psychosocial variables adopted, as well as the symptoms of CMD outcome (CMDS - Figure 3), some characteristics were identified, through the analysis of principal components that imprinted a profile of the participants based on the inverse relationship between CMD symptoms and perception about the social environment (NS, SR, SS, E, PH, Psy, SE - Figure 3). 

In the first collection phase, eight participants were identified who presented CMD symptoms with greater intensity and perception of the social environment. This behavior can be identified in the biplot plot by the dispersion of points (participants) around the CMDS variable (Figure 3 - Time A). In the same collection phase, another five participants were identified with lower scores for CMD symptoms and better perception of the social environment (Figure 3 - Time A).

In the second collection phase (Figure 3 - Time B) there was a change in the number of participants with CMD symptoms, with six having a greater variety of these symptoms and seven with lower variety. In the third (Figure 3 Time C -) and fourth collection (Figure 3 - Time D) phases, the number of participants with more CMD symptoms was reduced to four, indicating a gradual improvement in this aspect, as well as an increase in the number of participants with better perception of the social environment.

### Longitudinal behavior of the variables

Regarding the effect of time on the psychosocial variables analyzed, a significant interaction between group and time was identified only for the physical domain of QoL, which indicated the existence of a combined effect of time and groups on the participants’ perception in relation to the domain, that is, in the different phases evaluated (A, B, C and D), significant differences were observed for both groups: the women in Group 1 (more CMD symptoms) had their perception of the quality of this domain worsened overtime and those in Group 2 (better perception of the social environment) had it improved (Table 2). 

In turn, regarding the psychological domain of QoL, there was no significant interaction between group and time, although an isolated effect of time and group was identified, that is, in the different phases evaluated, Group 1 (more CMD symptoms) differed from Group 2 (better perception of the social environment) and, in both groups analyzed, the perception regarding quality of this domain increased over time (Table 2). 

In the social relationships and environment domains of QoL, as well as the indicators of satisfaction with social support, self-efficacy and CMD symptoms, there was a significant effect only for the group, that is, regardless of the phase analyzed, Group 1 (more CMD symptoms) differed from Group 2 (better perception of the social environment). The number of supporters was not significant (p>0.05) when groups 1 and 2 were analyzed in terms of the four phases analyzed (Table 2).

**Table 2:** Univariate Analysis of variance (ANOVA) with means and Tukey’s test (n=13). Ribeirão Preto, SP, Brazil, 2020-2021

Time	Physical^*^			Psychological^*^			Social Relationships^*^			Environment^*^		
	Group 1	Group 2	Mean	Group 1	Group 2	Mean	Group 1	Group 2	Mean	Group 1	Group 2	Mean
A	45.83 A b	64.29 A a	55.06	49.30	64.58	56.94 B	62.50	66.67	64.58 A	47.40	67.19	57.29 A
B	30.95 A b	81.55 A a	56.25	53.47	75.00	64.24 AB	61.11	84.72	72.92 A	41.15	74.48	57.82 A
C	40.48 A b	75.00 A a	57.74	46.53	75.70	61.11 AB	51.39	84.72	68.06 A	39.06	70.32	54.69 A
D	36.90 A b	83.93 A a	60.42	54.17	86.81	70.49 A	65.28	79.17	72.22 A	38.54	72.92	55.73 A
Mean	38.54	76.19		50.87 b	75.52a		60.07 b	78.82a		41.54 b	71.23 a	
*ANOVA*												
Group	9.25^†^			6.18^†^			5.87^†^			10.12^†^		
Time	2.01^‡^			4.12^†^			2.36^‡^			1.22^‡^		
Group*Time	4.79^†^			2.22^‡^			2.01^‡^			2.09^‡^		
CV%^§^ (a)	32			21			20			22		
CV% (b)	9			11			15			8		
*Time*	*Satisfaction with the support* ^*^			*Number of supporters* ^*^			*Self-efficacy* ^*^			*CMD symptoms* ^||*^		
	*Group 1*	*Group 2*	*Mean*	*Group 1*	*Group 2*	*Mean*	*Group 1*	*Group 2*	*Mean*	*Group 1*	*Group 2*	*Mean*
A	4.25	5.61	4.93 A	6.00	6.33	6.17 A	30.17	34.33	32.25 A	12.67	7.33	10.00 A
B	4.92	5.86	5.39 A	5.67	5.00	5.33 A	29.17	36.33	32.75 A	13.17	6.50	9.83 A
C	4.08	5.89	4.99 A	4.67	5.00	4.83 A	25.50	36.17	30.83 A	12.33	6.00	9.17 A
D	4.28	5.92	5.10 A	4.83	4.83	4.83 A	25.67	37.17	31.42 A	11.17	6.00	8.58 A
Mean	4.38 b	5.82 a		5.29 a	5.29 a		27.63 b	36.00 a		12.33 a	6.46 b	
*ANOVA*												
Group	6.79^†^			1.87^‡^			7.12^†^			5.58^†^		
Time	2.34^‡^			1.76^‡^			2.02^‡^			1.11^‡^		
Group*Time	2.62^‡^			1.58^‡^			2.21^‡^			1.11^‡^		
CV% (a)	15			26			15			39		
CV% (b)	4			12			6			14		

* Data were transformed (Box-cos) - The rounded lambda (λ) values of the Box-cos transformation for the data at different times (A, B, C and D) are as follows: Physical: 0.00; 0.50; 0.50; 0.50; Psychological: 0.00; 0.00; 0.50; 0.50; Social relationships: 0.00; 0.00; 1.00; 1.00; Environment: 0.00; 0.50; 0.50; 1.00; Satisfaction with the support: 0.50; 0.50; 1.00; -1.00; Number of supporters: -1.00; 0.50; -1.00; 0.50; Self-efficacy: 0.50; 0.50; 0.50; 0.50; CMD symptoms: 0.50; 0.50; 0.50; 0.00; λ = 0.50 (transformation used: square root); λ = 0.00 (transformation used: natural log); λ = 1.00 (no transformation); λ = -1.00 (inverse transformation: transformed value = 1 / original value), ^†^significant at 5%; ^‡^not significant (p>0.05) by Tukey’s test; ^§^CV% = Coefficient of Variation; ^||^CMD = Common Mental Disorder

## Discussion

The sociodemographic profile of the studied sample emphasized the participants’ conditions of social vulnerability. As shown in the results, two reported family incomes of more than two minimum wages, four had paid work and ten met the criteria for receiving emergency aid, which denotes a precarious living condition that was certainly intensified during the pandemic period, in view of the exponential increase in unemployment and the cost of living, which lead to even greater difficulties acquiring basic items by the poorest people^12^. In addition, studies carried out with settlement populations identified that food and financial insecurity was mentioned by the participants^13^ ^-^ ^15^, that is, the lower-income population was the one that suffered the most from the impacts of the pandemic^11^.

The fact that most of the participants declared themselves as black-skinned highlights the complexity of the gender, race and class intersection that significantly exacerbates the inequalities and vulnerabilities experienced by these women. In addition to that, many of them were home providers themselves and the main caregivers of children and aged people, exposing them disproportionately to the negative consequences of the pandemic, factors also evidenced in previous studies^9^ ^-^ ^10^ ^,^ ^12^. 

In view of the above, it is important to highlight solidarity actions by civil society itself, Non-Governmental Organizations and Brazilian Universities, which gained strength in the pandemic period, with a view to minimizing the negative consequences for people in situations of social vulnerability, such as donations of food, clothing, medications, hygiene items and masks, as well as educational campaigns to raise awareness about the risks of the disease and the importance of protective measures^28^ ^-^ ^30^. 

These initiatives were also given in response to the absence of effective governmental policies to fight against the pandemic, both in terms of public health and economics. Although emergency aid was instituted as a way of repairing such harms and as a result of society’s pressure and mobilization, the process in the Brazilian context took a long time and faced several problems in its implementation, namely: difficulties of the population carrying out the registration and having access to digital technologies to acquire the aid; long lines at bank branches that generated crowding; and precarious human resources, among others^31^, which possibly increased concern, insecurity and fear, considerably affecting the overall health of this group, making it even more vulnerable, even to infection by the severe acute respiratory syndrome coronavirus 2 (SARS-CoV-2).

Therefore, the 30.8% prevalence of infected people in the studied sample stands out, which is certainly related to such situations^4^ ^,^ ^8^. In view of the aforementioned aspects, it is inferred that the impacts of such morbidity may have been even worse when considering these women’s possible apprehension in contaminating other family members, especially considering the fact that they are the main caregivers, as previously reported. In addition to that, the positive diagnosis results in distancing from work activities, which may consequently have affected their incomes with paid jobs. Furthermore, the possible sequelae may have had a substantial impact on their quality of life, as well as negatively affect their health, as pointed out by previous studies^1^ ^,^ ^12^. 

In the results, it was identified that the women were well-discriminated between them in psychosocial terms, especially with regard to the perception of the social environment (environmental and relational aspects). In this sense, those who perceived this environment more positively were less vulnerable to emotional distress in the periods analyzed. This result corroborates a previous study that evaluated the impacts of COVID-19 on women’s mental health in Hong Kong and identified that those with better QoL and self-efficacy perceptions presented negative emotional symptoms to a lesser extent^12^. Considering that mental health encompasses complex and subjective issues such as the expression of emotions, communication and relationship resources and bargaining power in different life spheres, it is understood that strategies which emphasize promotion of these factors are extremely important and should be prioritized in the elaboration of health care plans.

It is worth noting that, in the cluster analysis, satisfaction with the support was identified as an important component for discrimination of the groups. However, the number of supporters exerted no significant effect on the outcomes considered. It is understood that this result can reflect the role of moderation and not necessarily the direct effect of the social support variable, as pointed out in previous studies, especially in those that observed perceived stress as outcome^32^ ^-^ ^33^. In addition to that, research studies on social support tend to consider the supporters’ attributes (family, friends, neighbors, others), the type of support (emotional, informational, instrumental) and the level of satisfaction with it (satisfied, dissatisfied)^33^ ^-^ ^34^ to a greater extent than the number of supporters itself. On the other hand, a previous study in which the number of supporters was evaluated also identified that the satisfaction item was more determinant of psychosocial protection than the number of supporters^35^. In this sense, the current result emphasizes the importance of satisfaction with the support as a construct to be prioritized in the implementation of mental health promotion strategies.

In relation to the impact of the different phases of the pandemic on the psychosocial aspects analyzed, the physical and psychological domains of QoL presented significant results.

In the psychological domain, there was an increase in scores over time in the sample as a whole. This result differs both from what was expected for a pandemic period and from the results of previous studies carried out with the general population of different countries^36^ ^-^ ^38^. Thus, in relation to the current study, it is worth noting the following: firstly, the specificities of the sample in terms of gender and social condition; secondly, the long-term character in relation to longitudinality and, also, the fact that data collection took place via telephone contacts. 

Regarding the specificities of the sample, it is hypothesized that, while the psychological domain encompasses issues related to positive and negative feelings, self-esteem, body image and appearance, thoughts, learning, concentration, memory and personal beliefs^21^, emotions are influenced by multiple factors both related to the aspect of more private life and to the social and community context. In this sense, considering the community organization of the settlements, as people face a situation of public calamity as lasting as the COVID-19 pandemic, relationships tend to strengthen and, consequently, expressions of affection, to intensify, which can result in a better perception in this regard.

In relation to the adoption of several data collection moments in the follow-up, it is understood that this methodological option may also have interfered in the result, as it made it possible to monitor, in a more accurate way, the oscillations of the scores in the different periods. Regarding the data collection modality, the results also suggest a possible effect of the interaction by the main author, who carried out data collection with the participants, as the fact that they felt accompanied over time, as well as the certainty that they would receive the call at pre-established moments may have emotionally influenced them in some way. 

Among the women who presented a more positive perception of the social environment (Group 2), an increase in the physical domain scores was identified during follow-up, while in the others (Group 1) there was a reduction in them. This QoL domain includes questions about pain and discomfort, energy and fatigue, sleep and rest, mobility, activities of daily living, dependence on medication or treatments, and work capacity^21^. Thus, considering the high prevalence of COVID-19 in the sample studied, the results of decreased QoL in this domain among the most vulnerable women can be considered unsurprising.

It is important to note that access to Brazilian health services for the entire population during the pandemic period was hampered due to canceled consultations and exams and limited care, only available in emergency cases. In the specific case of the sample studied, there was an aggravating factor, as the health unit located in the settlement was closed and the professionals were relocated to the unit in the urban area. Thus, access was made even more difficult both due to the pandemic issue and to the limitations already imposed and to the distance and displacement difficulty. 

Worse physical health outcomes also reflect issues related to the vulnerability and poverty cycle that further perpetuates and expands life- and health-threatening conditions. In this sense, the poorest population, with more precarious access to health services and who are unable to carry out the necessary follow-ups, generally have worse health outcomes, with negative consequences for work, income and quality of life in general^4^ ^-^ ^5^ ^,^ ^7^ ^-^ ^8^. 

Although the other aspects did not exert a significant impact over time, a well-demarcated division of groups was identified, so that women with multiple CMD symptoms also had worse results in terms of the psychosocial aspects analyzed during the entire follow-up period. It should be noted that, in general, previous studies report that large-scale catastrophes are commonly accompanied by worsening in the population’s mental health conditions^3^ ^,^ ^5^ ^,^ ^38^; therefore, groups already vulnerable for other reasons should have additional priority in public health and social protection policies, especially during these periods.

In this sense, it is understood that settled women are an extremely important group to consider when thinking about policies and strategies for health promotion in general. This is because, in addition to vulnerability to socioeconomic, racial and gender issues, we must add insecurity regarding housing and the difficulties arising from living in a settlement, such as food insecurity, unemployment, and precarious access to health, education, sanitation and transportation services^13^ ^-^ ^15^. 

In addition, the agricultural work developed by these women requires greater physical effort, which consequently affects their functional capacity, an item from the physical domain. Furthermore, this work burdens mental health, as production instability generates concerns, fear and anxiety about their livelihood, which were intensified in the pandemic period, as reported by some participants.

The findings of the current study showed that, despite this overlapping of vulnerabilities, the difference in the perception of the social environment proved to be a protective factor in relation to emotional distress, which has important implications in terms of the clinical practice, as health actions in general, which also make it possible to improve the users’ social environment, may exert an important impact on their mental health outcomes.

Thus, in terms of recommendations for the practice, in addition to the issues of improving the physical and psychological domain already discussed, the relevance of strategies focused on social support and self-efficacy of vulnerable women stands out. Regarding social support, its role as a protective factor for better results in mental health issues has been consolidated in the literature^35^ ^,^ ^39^ ^-^ ^40^. As it is an individual’s belief in their ability to deal with a wide variety of demands and face new situations, self-efficacy has also been associated with positive mental health outcomes^12^ ^,^ ^40^. 

In addition to that, previous studies specifically developed during the pandemic period have identified that social support feeds back the perception of self-efficacy, as a quality support network contributes to sharing experiences, which in turn can result in diverse information, self-knowledge building and individual understanding, in addition to access to different coping strategies, management and emotional venting^40^ ^-^ ^42^.

These factors become even more important when considering the fact that previous research studies indicate that the settled women’s mental distress is intensified due to the fact that they often have to silence their feelings because they do not feel welcomed to share them so much with their support network and with health professionals^13^ ^-^ ^14^. Thus, it is understood that, since health professionals, specifically nurses, are well positioned for mental health care, attention to such factors for the elaboration of care plans is essential, mainly in relation to care for settled women in the pandemic context.

In light of the foregoing, the importance of future studies considering socioeconomic, racial, cultural and gender specificities is emphasized, in order to expand the body of evidence that can better support the development, implementation and evaluation of social protection strategies, health promotion and prevention among the most vulnerable populations. In addition, it is recommended to use qualitative data to better understand the perception of these populations in relation to the psychological, subjective, complex and relational aspects of mental health in an integrated way, especially during periods of crises and pandemics when vulnerabilities are accentuated.

The study limitations refer mainly to the size and specificity of the sample, which limits generalization of the results, as well as to the fact that the data are self-reported. However, the importance of longitudinal studies developed in the pandemic context is highlighted, despite the countless challenges for collecting primary data in social isolation periods. In addition to that, studies on specific groups of women are extremely relevant for expanding the analytical spectrum in terms of health needs, care implementation and improvement of public policies and measures for health care and social protection, especially in low- and middle-income countries. 

## Conclusion

The current study aimed at analyzing the psychosocial impacts of the COVID-19 pandemic among Brazilian women from rural settlements. The sample was characterized by different vulnerability and poverty markers, exacerbated by the profile of rural workers who live with countless economic, social and health insecurities, even more intensified by the impacts of the pandemic.

Despite the numerous risk factors, it was identified that, in general, such women presented favorable results for the Quality of Life psychological domain. In addition to that, both physical health and the social environment proved to be protective factors in relation to the mental disorder symptoms, emphasizing the importance of the synergy of the different constructs in the elaboration of care plans, as well as the urgent need for more effective and specific public policies targeted at certain population groups, especially in times of crisis and pandemics.
